# Interpreting Alzheimer’s disease clinical trials in light of the effects on amyloid-β

**DOI:** 10.1186/alzrt244

**Published:** 2014-03-12

**Authors:** Jeremy H Toyn, Michael K Ahlijanian

**Affiliations:** 1 Bristol-Myers Squibb Research and Development, Neuroscience Biology, 5 Research Parkway, Wallingford, Connecticut 06492, USA

## Abstract

The failure of several potential Alzheimer’s disease therapeutics in mid- to late-stage clinical development has provoked significant discussion regarding the validity of the amyloid hypothesis. In this review, we propose a minimum criterion of 25% for amyloid-β (Aβ) lowering to achieve clinically meaningful slowing of disease progression. This criterion is based on genetic, risk factor, clinical and preclinical studies. We then compare this minimum criterion with the degree of Aβ lowering produced by the potential therapies that have failed in clinical trials. If the proposed minimum Aβ lowering criterion is used, then the amyloid hypothesis has yet to be adequately tested in the clinic. Therefore, we believe that the amyloid hypothesis remains valid and remains to be confirmed or refuted in future clinical trials.

## Introduction and scope

Alzheimer’s disease (AD) is a devastating and costly disease accounting for 50 to 80% of senile dementia cases. Worldwide, over 35 million people have dementia and the number is projected to double in the next 20 years [1]. Current treatments for symptoms have marginal benefits, and none treat the disease itself. A key hallmark of AD brain pathology is the accumulation of amyloid plaques. These consist largely of amyloid-β (Aβ) peptide, which is formed through proteolytic cleavage of amyloid precursor protein (APP) by two proteases: β-site APP-cleaving enzyme (BACE) and γ-secretase. Rare mutations in APP and the catalytic subunit of γ-secretase, presenilin, cause inherited forms of AD (familial AD (FAD)) with accelerated age of onset. In addition there are genetic risk factors, such as apoE4 and the APP Iceland mutant, that respectively increase or decrease AD risk. These genetic polymorphisms are all associated with changes in the production of Aβ, or changes in the relative amount of the more neurotoxic 42 amino acid form of Aβ, ‘Aβ42’ [2]. Thus, genetic and pathological evidence has converged on the amyloid hypothesis of AD, proposing that accumulation of Aβ is neurotoxic, leading to neuron loss, dementia and death [3,4]. Accordingly, major approaches to AD drug development over the past two decades have focused on lowering Aβ - for example, by inhibition of BACE or γ-secretase, or by the use of therapeutic antibodies to neutralize or enhance clearance of Aβ. Unfortunately, several clinical trials based on these approaches have been unsuccessful, raising the question of whether failure was due to insufficient target engagement, trial design, or the amyloid hypothesis. Here we address the target engagement question: what is the minimum extent of Aβ lowering sufficient for significant cognitive benefit in AD patients? And has this level of target engagement yet been achieved in patients for sufficient trial duration?

## Evidence in humans for the effect of changes in amyloid-β production

Human genetic evidence suggests that modest changes in Aβ production are associated with a significant impact on AD. FAD mutants in which the APP gene is duplicated increase the gene dosage of APP by 50%, implying increased Aβ production [5]. This suggests that a 33% decrease of Aβ production in affected individuals would result in Aβ production rates equivalent to that of normal healthy individuals. A similar situation of 50% increased APP gene dosage due to trisomy 21 is associated with >50% increase in APP mRNA expression, and may contribute to early onset AD in Down’s syndrome [6]. In sporadic (late onset) AD, a 30% decreased clearance of Aβ was reported in AD subjects, based on data using a heavy isotope labeling method [7]. In contrast to the FAD mutants, one rare APP mutant was associated with decreased incidence of AD [8]. In cell cultures overexpressing this mutant, BACE cleavage of the mutant APP was decreased by 50%, thereby decreasing Aβ production. This result implies that Aβ production in heterozygous individuals would be decreased by about 25%, although direct measurements of Aβ production in these individuals have not been reported. Thus, accumulating evidence suggests that relatively modest changes in Aβ, perhaps as little as 25% change over a sufficient period of time, can have a significant impact on AD.

In addition to the association of decreased Aβ levels with decreased disease risk, increased production of Aβ42, relative to other Aβ peptides, is associated with earlier age of disease onset. Studies of Aβ production in cell cultures expressing presenilin FAD mutants showed that the relative amount of Aβ42, measured as an Aβ42/Aβ40 ratio, was inversely correlated with age of onset [9,10]. To a first approximation, an earlier age of onset by 1 year was associated with a 1% increased Aβ42/Aβ40 production ratio, as measured in cell cultures. Another study reported an FAD mutant in which Aβ40 was selectively decreased without change in Aβ42, thus further emphasizing the role of the ratio [11]. Aβ42/Aβ40 production ratios are more challenging to measure *in vivo*, and require methods that circumvent the confounding effects of Aβ aggregation and degradation in the brain. The recent stable isotope labeling study by Potter *et al*. [12] reported that presenilin FAD subjects had a 24% increased Aβ42/Aβ40 production ratio, and selectively increased Aβ42 clearance in subjects with amyloid deposits. This is consistent with the amyloid hypothesis; increased Aβ42 production leads to increased aggregation in the brain, thereby decreasing the amount of Aβ42 transported into the cerebrospinal fluid (CSF). This results in the counterintuitive situation in which increased Aβ42 levels in brain lead to decreased Aβ42 in CSF. In an earlier report using the stable isotope labeling method, sporadic AD patients (who were not FAD carriers) had decreased clearance in Aβ42 and Aβ40 of 30% and 26%, respectively, but no difference in production rates relative to age-matched controls [7]. Clearly, more studies are required to understand differences in Aβ dynamics between different genotypes and stages of disease, but thus far it appears that increases in either total Aβ or Aβ42 production can accelerate disease onset. In contrast to presenilin FAD mutants, APP FAD mutants were reported to increase Aβ38 production, in addition to Aβ42, relative to other Aβ peptides, and *in vitro* results raised the possibility that Aβ38 may also contribute to aggregation and neurotoxicity [13]. Thus, small changes, most likely less than 25%, in the ratios of Aβ peptides are associated with profound changes in AD risk and age of onset. The human evidence described in the above section is summarized in Table 1.

**Table 1:** Alzheimer’s disease and human Aβ levels

**Disease state**	**Affect on Aβ**	**Type of study**	**Reference**
APP gene duplication	50%↑ production inferred	Gene copy number in patients	[5]
Trisomy 21	50%↑ production inferred	mRNA levels in patients	[6]
Protective APP allele	25%↓ production	Cell culture	[8]
Presenilin FAD	Aβ42/Aβ40 1%↑ per year earlier age of onset	Cell culture	[9,10]
Presenilin FAD	Aβ42/Aβ40 24%↑ clearance	Patient CSF samples	[12]
Sporadic AD	Aβ42 30%↓ clearance; Aβ42 26%↓ clearance	Patient CSF samples	[7]
APP FAD	Aβ42↑ and Aβ38↑ production	Cell culture	[13]

Aβ, amyloid-β; AD, Alzheimer’s disease; APP, amyloid precursor protein; CSF, cerebrospinal fluid; FAD, familial Alzheimer’s disease.

## Evidence from Alzheimer's disease mouse models for the effect of changes in amyloid-β levels on cognition

APP transgenic (TgAPP) mice are engineered to overexpress human APP, and in most cases exhibit Aβ-dependent pathology and cognitive deficits. Multiple genetic and pharmacological methods have been used to explore Aβ changes in these models. The soluble pool of Aβ responds rapidly to changes in Aβ production, whereas amyloid plaque-associated Aβ accumulates slowly with age, and does not respond acutely to changes in Aβ production. Therefore, we first considered studies that reported measurements of soluble Aβ-lowering and associated cognitive outcomes in TgAPP mice (Table 2).

**Table 2:** Aβ-lowering cognitive benefit in TgAPP mice

**Aβ-lowering method**	**Brain Aβ lowering (%)**	**Observed functional benefits**	**Mouse strain**	**Reference**
BACE1-/- KO	>90%	Contextual fear conditioning, Morris water maze, social recognition	Tg2576; 5xFAD	[14-16]
BACE+/- KO	12% in young mice	Contextual fear conditioning, conditioned taste aversion	PDAPP; 5xFAD	[17-20]
Presenilin conditional forebrain KO	75%	No benefit (novel object recognition in 3- to 6-month-old mice)	APP [V717I]	[25]
Presenilin conditional forebrain KO	55% in young mice	Contextual fear conditioning and Morris water maze in young but not old mice	APP J20	[26]
TgAPP conditional allele	≥70% new Aβ; no effect on steady-state Aβ42 levels	Two trial Y maze; plus water maze; radial arm water maze	Repressible TgAPP	[27]
Cystatin C KO	40% (Aβ); 60% (Aβ42) in young mice	Morris water maze	APP J20	[28]
GRL-8234 (BACEi)	35-50% plaque after 7 months	Morris water maze	Tg2576	[31]
TAK-070 (BACEi)	20% plaque after 7 weeks	Y-maze, Morris water maze, novel object recognition	Tg2576	[29]
Trihydroxychalcone (BACEi)	50-60% plaque after 106 days	Morris water maze	APP-PS1	[28]
DAPT (GSI) single dose	25% at 8 hours	Contextual fear conditioning	Tg2576	[32]
DAPT (GSI) repeat dose	35% after 4 days	Morris water maze	Ts65Dn	[34]
Begacestat (GSI) single dose	25-35% at 4 hours	Contextual fear conditioning	Tg2576	[33]
Semagacestat/ LY450139 (GSI)	No change 1 mg/kg; 25-30% 10 mg/kg	Y maze benefit at 1 mg/kg after single dose; no benefit at 10 mg/kg or 8-day repeat dosing	Tg2576	[35]
Avagacestat/ BMS-708163 (GSI)	No change 1 mg/kg; 25-30% 10 mg/kg	Y maze benefit at 1 mg/kg after single dose; no benefit at 10 mg/kg or 8-day repeat dosing	Tg2576	[35]

Aβ, amyloid-β; BACE, β-site APP-cleaving enzyme; BACEi, β-site APP-cleaving enzyme inhibitor; GSI, γ-secretase inhibitor; KO, knock out; TgAPP, APP transgenic.

BACE1 knock out (KO) mice exhibited a range of Aβ lowering from 12% for heterozygous to >90% for homozygous animals, with cognitive benefits in multiple types of cognitive assays [14-20]. In contrast, ablation of γ-secretase caused developmental abnormal or lethal phenotypes, and conditional KO (cKO) alleles of presenilin or nicastrin caused neurodegeneration and memory deficits in wild-type mice [21-24]. Thus, it is hardly surprising that presenilin cKO did not consistently show cognitive benefits in TgAPP mice despite Aβ lowering in the 55 to 75% range [25,26]. For γ-secretase ablation, it is possible that any benefit of Aβ lowering is confounded by deficits caused by loss of other functions of γ-secretase, such as Notch receptor activation. In addition, the restriction of the presenilin cKO allele to the forebrain may not have targeted Aβ lowering to the optimal anatomical location for benefit in TgAPP. A repressible TgAPP allele has been used to control Aβ synthesis in TgAPP mice [27]. In this study, aged plaque-bearing mice were fed doxycycline to repress TgAPP expression, implying a corresponding decrease in newly synthesized Aβ. Cognitive improvement was detected after 7 days, and yet no detectable lowering of transgene-derived soluble Aβ42 was apparent, presumably due to equilibrium of soluble Aβ42 with plaque Aβ42. Aβ can also be decreased by cystatin C KO, which increases Aβ clearance via increased cathepsin B protease activity. Cognitive benefits in cystatin KO mice were associated with Aβ lowering of about 40% in young plaque-free TgAPP mice [28].

Improved cognition in TgAPP mice chronically dosed with BACE inhibitors (BACEis) GRL-8234, TAK-070 and trihydroxychalcone was associated with amyloid plaque lowering in the 20 to 60% range, but no evidence of decreased Aβ production was reported [29-31]. TgAPP mice given single doses of the γ-secretase inhibitors (GSIs) DAPT, begacestat, semagacestat, and avagacestat showed cognitive improvements with Aβ lowering in the range 0 to 35% [32-36]. The effect of a single dose is noteworthy because it implies an acute role of newly synthesized Aβ in cognitive impairment. In the study by Mitani *et al*. [35], a 1 mg/kg single dose of semagacestat or avagacestat improved Y maze performance, although decreased Aβ was only detectable at higher doses. However, 8-day repeat dosing at 1 mg/kg did not improve Y maze performance in TgAPP mice, and actually impaired Y maze performance in wild-type mice. Thus, like the presenilin cKO allele, it appears any benefit of Aβ lowering in TgAPP mice may have been confounded by other deficits resulting from γ-secretase inhibition, in this case proposed due to accumulation of APP β-CTF fragment [35].

Selective lowering of Aβ42 is of therapeutic interest because of increased Aβ42 in FAD mutants, the evidence that Aβ42 is the earliest deposited species [37], and the cognitive disruption caused by Aβ42 aggregates in animal models [38,39]. Furthermore, *in vitro* studies have shown that Aβ42 aggregation is inhibited by Aβ40 [40-42], and also by Aβ37 and Aβ38 [43], suggesting that the shorter peptides are capable of interfering with the amyloid cascade. A variety of genetic and pharmacological methods have been used to selectively alter Aβ42 levels *in vivo*. An increased Aβ42/Aβ40 ratio enhanced aggregation and neurotoxicity *in vitro* and caused memory deficits after a single intraventricular injection in wild-type mice [44]. A presenilin mutant that selectively lowered Aβ40 exacerbated plaque deposition in TgAPP mice, implicating the Aβ42/Aβ40 ratio *per se*[45]. In another *in vivo* approach, novel Tg-Aβ42 and Tg-Aβ40 transgenes were used for selective expression of Aβ42 or Aβ40, respectively. Selective expression of Aβ40 was shown to interfere with Aβ plaque accumulation in Tg-Aβ42 and TgAPP mice [46]. Remarkably, however, Tg-Aβ42 and Tg-Aβ40 mice exhibited no cognitive defects in a range of tests, indicating that overexpression of Aβ was insufficient for neurotoxicity in this model [47]. As mentioned above, a cystatin C KO in TgAPP mice ameliorated cognition associated with 40% overall lowering of Aβ peptides; however, this was also in the context of 33% relative lowering of Aβ42 [28].

γ-Secretase modulators (GSMs) include a variety of small molecules that target γ-secretase, causing decreased Aβ42 and increased production of one or more shorter peptides such as Aβ37, -38, or -39 [48]. Thus, GSMs have an essentially opposite effect to FAD mutants. The GSM EVP-0015962 improved cognition in TgAPP mice after a single dose that caused a 50% decrease in Aβ42 [49]. CHF5074 improved cognition after chronic dosing in TgAPP mice with no discernable Aβ42 lowering, but it seems probable that the cognitive effect was not related to the GSM activity of this compound, which is of very low potency [50-53]. TgAPP mice given single doses or 8-day repeat doses of GSM-2 at ≥0.1 mg/kg showed improved Y maze performance [35], although Aβ42 lowering, of 20% and 30%, was detected only at the higher doses of 1 and 3 mg/kg, respectively [35,36]. The GSMs JNJ40418677 and ‘compound 4’ exhibited Aβ42 lowering activity in the 40 to 50% range, but cognitive effects were not reported. However, long term dosing of these compounds did decrease Aβ plaque accumulation [54,55]. Thus, accumulating evidence suggests that decreased Aβ42 relative to shorter Aβ production affects the amyloid cascade and improves cognitive performance in TgAPP models, as summarized in Table 3.

**Table 3:** γ-Secretase modulators and selective Aβ42-lowering benefit

**Selective Aβ42-lowering method**	**Brain Aβ42 lowering (%)**	**Observed cognitive or pathological benefits in deficient Tg mouse**	**Mouse strain**	**Reference**
ICV injection of preaggregated Aβ42/Aβ40	Aβ42/40 3:7 ratio; 1:9 ratio inactive	Passive avoidance and contextual fear conditioning	Wild type; intraventricular Aβ administration	[44]
BRI-Aβ40 and BRI-Aβ42 transgenes	50-400% increased Aβ40 (decreased 42/total ratio)	60-90% decreased plaque; improved survival; however, these mice exhibited no Aβ-dependent cognitive phenotypes	Tg2576 and Tg-Aβ40	[46,47]
EVP-0015962	50% after single 30 mpk dose	Contextual fear conditioning, gliosis 75% plaque load, after 50 weeks at 60 mpk/day	Tg2576	[49]
CHF5074	No significant change (4–9 month treatment)	Contextual memory, 50-75% decreased plaque burden, astrogliosis, synaptophysin levels, neurogenesis	Tg2576	[50-53]
GSM-2	0-30% at 0.1-3 mpk, respectively	Y maze improvements at 0.1-3 mpk in mice aged 5.5 months	Tg2576	[35]
GSM-2	50-60% nascent Aβ 2 hours after 10 mpk	Y maze and plaque pathology in mice aged 10–18 months	Tg2576	[36]
JNJ40418677	50% max lowering 30 mpk single dose	Up to 96% decreased plaque area and number after 7 months at 120 mpk/day	Tg2576	[55]
Compound 4	40% decrease 100 mpk single dose	48-76% decrease of plaque Aβ after 7 months at 50 mpk/day	Tg2576	[54]

Aβ, amyloid-β; ICV, intracerebroventricular; mpk, mg/kg; Tg, transgenic.

The interpretation of the evidence linking Aβ lowering and cognitive benefits in animal models should take several factors into account, including the mechanism by which Aβ lowering was achieved, and the possibility of confounding toxicity, as well as the observed change in Aβ levels. For example, sustained Aβ lowering is likely to be more impactful than transient Aβ lowering. For genetic methods of Aβ ablation, measurement of soluble Aβ levels at a single time point represents the overall sustained level of Aβ lowering. For small molecules, however, Aβ lowering data often refer to a single optimal time point after dosing, which can be several-fold greater than the average extent of Aβ lowering across the dosing interval. In addition, the form of Aβ measured should be considered. Many studies, including immunization approaches, have reported cognitive benefits associated with decreased plaque Aβ. Decreased plaque Aβ is a downstream endpoint, and is not a direct readout for decreased Aβ production or neurotoxic forms of Aβ. Nevertheless, such studies give further evidence of the link between the amyloid cascade and cognition [56].

Thus, taking into consideration a wide range of studies in TgAPP mice and human genetics, relatively modest decreases in Aβ, of about 25%, are associated with cognitive benefits (Tables 2 and 3). Therefore, we propose that sustained Aβ lowering of 25% using any method tolerated for a sufficient period of time in patients represents a reasonable minimal objective. While this criterion is proposed as a minimal objective, an optimal therapeutic will provide the flexibility to probe a range of Aβ-lowering activity, including nearly complete lowering, in order to understand the relationship between Aβ lowering and efficacy. Nevertheless, greater than 25% may not be achievable by some compounds, and consequently setting the bar too high could result in lost opportunities. Lowering of Aβ by approximately 25% therefore sets a reasonable starting point for the minimum level of pharmacodynamic effect to justify efficacy trials in AD patients.

## Demonstration of amyloid-β lowering in recent clinical trials

If the preceding arguments are valid, then a pressing question is whether the recent, late stage clinical studies achieved the 25% Aβ lowering criterion. Before this question can be addressed, however, two antecedent questions require clarification; at what stage of AD might 25% Aβ reduction produce efficacy, and what form of Aβ should be targeted for 25% reduction.

What is the relationship between the extent of Aβ lowering required for efficacy and disease stage? For example, does it escalate with disease progression - that is, is the requirement for Aβ reduction lower if intervention is earlier (predementia/presymptomatic), and greater if intervention is later in disease (mild to moderate)? Alternatively, is there some degree of Aβ lowering that will produce efficacy regardless of stage of intervention? Finally, is there a point in the disease process that is unresponsive to Aβ-directed therapies (for example, moderate to severe)? While clear answers to these questions will not be forthcoming until an efficacious agent is identified, there seems to be consensus in the field that earlier intervention is desirable [4,57]. This consensus is based on the long latency of measurable pathologic changes (changes in CSF Aβ and tau, plaque and tangle development, volumetric magnetic resonance imaging (MRI)) and the relatively late onset of cognitive symptoms [58-64]. Based on the hypothesis that earlier intervention is better, several clinical efficacy studies targeting pre-symptomatic AD patients are either underway or planned (for example, [65,66]). For the purposes of this review, we propose the minimum criterion of 25% Aβ lowering for clinical trials targeting early stages of the disease, namely predementia (mild cognitive impairment with biomarker evidence consistent with AD) and mild AD. The combination of cognitive symptoms with biomarkers such as CSF Aβ42, tau, volumetric MRI and amyloid positron emission tomography (PET) suggest that these are the earliest disease stages for which a diagnosis of AD or likely progression to AD can currently be confidently assigned (for example, [59,67,68]). However, even mild AD may be too late for initiating Aβ-lowering therapies given the latency between biomarker positivity and symptom onset. Therefore, the 25% criterion could also be considered when designing trials for presymptomatic AD.

Which form of Aβ should be targeted for 25% reduction in efficacy trials? The amyloid hypothesis currently states that soluble Aβ is the species most deleterious to neuronal viability and synaptic function. While the precise molecular identity of the most toxic Aβ species is debatable (for example, [69]), the number of independently reproduced reports implicating soluble Aβ as disruptive to normal function strongly suggests that this species plays a key role in the cognitive decline observed in AD.

If soluble Aβ is the key culprit in cognitive impairment, how can sponsors assess potential reduction of this species in humans in clinical trials? Currently the best reflection of soluble brain Aβ is CSF Aβ [70]. CSF Aβ is used to aid in the diagnosis of AD [71-73] and has been used as a target engagement biomarker by sponsors developing therapies that are intended to lower Aβ [74,75]. The latter studies are typically supported by substantial preclinical data sets demonstrating an understanding of the relationship between brain and CSF Aβ-lowering produced by an Aβ-targeting compound in more than one species. These preclinical studies have demonstrated close correspondence between brain and CSF lowering of Aβ produced by GSIs [33,76-79], GSMs [70,79,80] and BACEis [70,81-83] confirming that CSF Aβ can reflect brain Aβ. These preclinical data sets are subsequently used as the basis for pharmacokinetic/pharmacodynamic (PK/PD) modeling to aid in dose selection and for determining the time points to sample CSF in human studies. For example, the GSI avagacestat produced reductions in rat brain Aβ that were reflected by comparable reductions in CSF after acute administration [78]. Modeling of these data accurately predicted the human PK/PD relationship for reductions in normal healthy volunteer (NHV) CSF [78,84,85]. Furthermore these PK/PD relationships for Aβ lowering did not differ significantly between NHVs and AD patients [86]. Additional preclinical data sets followed by PK/PD modeling and data collection in humans have been reported for other classes of Aβ-lowering drugs, including GSMs (BMS, unpublished) and BACEis [82,83]. Thus, for all synthesis-inhibitor mechanisms studied in this way, there is substantial correlation between lowering of Aβ in brain and CSF in preclinical species. Furthermore, modeling the preclinical data for translation has faithfully predicted the PK/PD of CSF Aβ lowering in both NHVs and AD patients.

Nevertheless, the presence of plaques in patients presents a potential confound for interpreting or expecting changes in CSF Aβ in patients. There is still active debate regarding the role of amyloid plaques in producing the cognitive deficits observed in AD (for example, [87]). Neurons that are proximal to plaques display aberrant dystrophic neurites with disrupted trajectories indicative of synaptic dysfunction (for example, [88]) and plaques have been hypothesized to create and sustain neurotoxic microenvironments [89] and perturb mitochondrial function [90]. In patients, alterations in functional brain connectivity have been reported in plaque-bearing regions in cognitively normal subjects (for example, [91,92]). However, it remains unclear to what extent such proximal, plaque-associated dysfunction contributes to the global cognitive impairment observed in AD patients, particularly since cognitive function did not improve in a small number of patients with reduced plaque after treatment with AN1792 [93,94].

It has also been hypothesized that amyloid plaques are protective and serve as a mechanism for clearance of soluble amyloid species from the interstitial space (for example, [87]). Furthermore, under conditions in which soluble Aβ is decreased (for example, in the presence of a therapeutic that lowers soluble Aβ) there is speculation that the most recently plaque-associated Aβ may dissociate, re-attaining a soluble state in parenchyma and the potential to become toxic to neurons. Pre-clinically, measurements of interstitial Aβ suggest equilibrium between soluble and insoluble forms of Aβ [95] and a study that compared plaque removal by two antibodies that recognized either soluble or fibrillar Aβ demonstrated that only the fibril-preferring antibody decreased plaque load [96]. Finally, a recent study suggests that, in the presence of very low plasma Aβ, plaque volume does not change in Tg mice [97].

In the clinic, an examination of the PK/PD analyses comparing the CSF Aβ-lowering effects of avagacestat in AD patients and NHVs demonstrates little difference in these two populations, suggesting that the potential contribution of soluble Aβ derived from plaque may be modest [79,86]. Similarly, recent evidence from BACEi studies in AD patients and NHVs suggests that the potency for reducing Aβ peptides is equivalent in these human populations and that the fraction of CSF Aβ peptides that is not sensitive to BACE inhibition (and therefore may be derived from an alternative source, such as plaques) is quite small, ranging from 2 to 6% [98]. Furthermore, any association of soluble Aβ to plaques does not limit the ability to detect therapy-induced decreases in CSF Aβ in patients [86,99]. Thus, our view is that while soluble Aβ is likely to be in equilibrium with plaques [12,95], and some fraction of soluble Aβ will associate with plaques, the data reported to date suggest that plaques are unlikely to provide a significant supply of soluble Aβ to CSF. Therefore, the ability to detect Aβ lowering in the CSF of AD patients should not be confounded by the presence of plaques, especially if lowering has been demonstrated in healthy volunteers. Nevertheless, the Aβ PET ligands have a clear role in AD diagnosis and can be used as target engagement biomarkers for some potential therapeutics, including antibodies [100,101].

How have the different Aβ-lowering mechanisms fared in late stage clinical trials? A summary is provided in Table 4. Avagacestat was tested in both mild-moderate and pre-dementia patient populations without evidence of efficacy, but the pre-dementia study was discontinued prior to the planned completion. The acute and steady state lowering of CSF Aβ produced by avagacestat in NHVs was substantial, but tolerability declined at doses that lowered CSF Aβ by more than approximately 15% in NHVs and especially in AD patients [85,86]. Thus, the maximum tolerated Aβ lowering was less than the 25% minimum criteria proposed above.

**Table 4:** Cerebrospinal fluid Aβ lowering - summary of clinical trial results

**Name**	**Mechanism**	**Stage of development**	**NHVs**	**Patient population**	**Patient CSF Aβ**^**a**^	**Amyloid PET**	**Reference**
AN1792	Active vaccine	D/C (phase IIa)	NR	M-M	No change	NR	[115]
Bapineuzumab	Passive vaccine	D/C ( phase III (i.v.); phase II (s.c.))	NR	M-M	No change	Decrease	[74,116]
Solanezumab	Passive vaccine	Phase III, pre-sym	NR	M-M, mild	Total (40/42) - increased Unbound 42 - increased Unbound 40 - decreased	NR	[117,122]
Crenezumab	Passive vaccine	Phase I/II	NR	Pre-sym, FAD	NR	NR	[127]
Gantanerumab	Passive vaccine	Phase II/III	NR	M-M, pre-dem	NR	Decrease	[101]
IVIG	Anti-inflammatory	Phase III	NR	M-M	No change	NR	[126]
Tarenflurbil	GSM	D/C (phase III)	NR	M-M	NR	NR	[109]
Semagacestat	GSI	D/C (phase III)	No change	M-M	No change	NR	[102-104]
Avagacestat	GSI	D/C (phase II)	≥50% decrease	M-M	High dose: ~50% decrease Tolerated doses: ≤15% decrease	NR	[79,85,86]
LY2811376, LY2886721	BACE inhibitor	D/C (phase II)	≥50% decrease	M-M	NR	NR	[82]
MK8931	BACE inhibitor	Phase II	NR	M-M	≥80% decrease	NR	[99]

^a^Most advanced stage clinical trial. Aβ, amyloid-β; BACE, β-site APP-cleaving enzyme; CSF, cerebrospinal fluid; D/C, clinical development discontinued; FAD, familial Alzheimer's disease; GSI, γ-secretase inhibitor; GSM, γ-secretase modulator; i.v., intraventricular; M-M, mild to moderate Alzheimer’s disease; NHV, normal healthy volunteer; NR, not reported; PET, positron emission tomography; pre-dem, predementia; pre-sym, presymptomatic; s.c., subcutaneous.

None of the published clinical data for semagacestat disclose evidence for steady state lowering of CSF Aβ in either NHVs [102] or AD patients [103,104]. However, a stable isotope labeling kinetic (SILK) study did demonstrate acute inhibition of the appearance of newly synthesized Aβ in the CSF [105] and reduction of shorter forms of Aβ have been interpreted as target engagement [106,107]. While the SILK study provided evidence for target engagement and inhibition of Aβ synthesis for a short period of time after dosing, the lack of steady state lowering of CSF Aβ at tolerated doses suggests that semagacestat may not have lowered soluble brain Aβ to a significant degree in NHVs or AD patients. Phase III studies demonstrated that semagacestat was not efficacious but exacerbated the cognitive decline in treated patients [108]. Thus, for the two GSIs that have achieved late stage clinical development, neither have achieved 25% lowering of soluble CSF Aβ in AD patients at tolerable doses and both have failed in the clinic. Taken together, the avagacestat and semagacestat examples suggest that, compared with NHVs, AD patients may be more sensitive to any unintended effects of potential therapeutics.

A small number of GSMs have also been tested in both NHVs and AD patients. Tarenflurbil failed in phase III [109], but CSF Aβ lowering was not reported in humans and the ability of the compound to lower brain Aβ in preclinical species has been the subject of debate [110,111]. CHF-5074 lowers Aβ in mouse models of APP overexpression but only after chronic dosing (that is, there are no sub-acute effects of this compound on brain Aβ [51]) making PK/PD analyses challenging and calling into question the mechanism of Aβ lowering after chronic treatment. Nevertheless, this molecule has completed a small, 12-week phase II study in AD patients [112] and, while CSF Aβ was measured, no changes were reported. Several companies [113] have disclosed preclinical Aβ lowering data on GSMs and some of these publications include measurement of both brain and CSF and PK/PD analyses [80,81]. However, no clinical data have been released despite the disclosure of phase I studies sponsored by BMS (New York, NY, USA) and Eisai (Tokyo, Japan).

While clinical data for GSMs are scarce, there are excellent examples of preclinical PK/PD data sets generated with BACEis, with subsequent translation to clinical studies in one instance thus far [82,83]. Lilly (Indianapolis, IN, USA)has disclosed the most data, establishing a convincing relationship between brain and CSF lowering with subsequent PK/PD modeling and translation to humans [82]. Merck (Whitehouse Station, NJ, USA) has also disclosed clinical data with a BACEi [99]. The extent of lowering of CSF Aβ produced by both of these BACEis in NHVs and AD patients is unprecedented and can exceed 90%, suggesting that lowering of Aβ in the brain is substantial. Unfortunately, the lead Lilly molecule, LY2886721, produced hepatic adverse effects in AD patients (13 June 2013, Lilly press release) which forced termination of the phase II study. However, the phase II development of the Merck BACEi continues, suggesting that the hepatic issues produced by LY2886721 may be off-target, compound-specific and unrelated to BACE inhibition. Importantly, these data indicate that BACE inhibition is currently the most promising therapeutic modality to directly test the Aβ hypothesis of AD.

## Anti-amyloid-β antibodies and IVIG

More than a decade after the initial reports of positive effects on pathology and cognition produced by Aβ immunization in TgAPP mice [114], it is now well established that reduction of plaque volume and restoration of functional deficits in TgAPP mice can be achieved by both passive and active Aβ immunotherapy [56]. It was these findings in preclinical models that prompted clinical development of Aβ immunotherapy. However, the results from the late phase clinical studies assessing this modality have been predominantly negative. AN1792, an active vaccine, was discontinued in phase II due to meningoencephalitis [115] and produced a small increase in CSF Aβ. Two passive anti-Aβ immunotherapies, bapineuzumab and solanezumab, have completed phase III clinical studies. Intravenous administration of bapineuzumab failed [116], but a subcutaneous study continues. Solanezumab has completed two phase III studies with mixed results [117]. However, the data for solanezumab in mild AD patients were sufficiently encouraging to warrant an additional phase III study (3 July 2013, Eli Lilly press release).

What is the relationship of these results to the proposal that a minimum of 25% soluble Aβ reduction must be achieved to produce efficacy? Unfortunately, the answer to this question is unclear. Assessing the PK/PD of antibody therapy is more challenging than for small molecule therapy for several reasons, including antibodies that recognize multiple forms of Aβ (soluble, fibrillar), no direct measure of target engagement in brain (antigen-antibody complex), difficulty in assessing free antibody concentrations compared with total concentrations (that is, antibody bound to antigen), and a large pool of antibody in plasma that can exchange with brain antibody [118-120]. Preclinical studies assessing the effects of antibody therapy on CSF concentrations of Aβ are rare if not non-existent, leaving clinicians with very little data upon which to base the dose selection and dosing intervals required for efficacy. Nevertheless, in clinical studies for both solanezumab and bapineuzumab, assessments of CSF Aβ and tau were made. In the bapineuzumab phase II and III studies, no changes in CSF Aβ were detected and small decreases in both phospho-tau181and total tau were reported [71,72,116,121]. For solanezumab the picture is more complex. In the phase II study, total CSF Aβ40 and Aβ42 increased. Unbound Aβ42 also increased while unbound Aβ40 was non-significantly decreased [122]. The increase in total CSF Aβ was interpreted as evidence for central nervous system penetration of the antibody while the increase in CSF Aβ42 was suggested to be due to potential dissolution of amyloid plaques (see below). In the phase III studies, unbound Aβ40 decreased while unbound Aβ42 did not change compared to controls [117]. The increases in total CSF Aβ were interpreted as evidence for central nervous system penetration of the antibody while the changes in unbound Aβ peptides were suggested to be due to potential alterations in compartment equilibria (for example, central to peripheral or fibrillar to soluble). In summary, the Aβ immunotherapy data disclosed to date does not provide a clear picture on the effects on unbound, soluble CSF Aβ, suggesting that the utility of CSF Aβ as a target engagement biomarker for immunotherapy may be limited. Alternatively, the potential efficacy and biomarker effects of the antibodies may not have manifested due to limitations in dosing levels or frequency and the failure to achieve efficacious brain concentrations. For example, in contrast to small molecule therapy, the implications of these negative findings are difficult to interpret due to the lack of preclinical analyses that define a relationship between antibody exposure, brain and CSF Aβ, and functional measures of efficacy such as synaptic and cognitive measures.

IVIG is a purified preparation of human immunoglobulins that has been used therapeutically for immune-deficiency disorders. Based on preclinical data and the hypothesis that IVIG would provide a source of anti-Aβ antibodies and possibly anti-inflammatory activity [123,124], IVIG has been evaluated through phase III clinical trials. While reductions in total CSF Aβ were reported for small pilot studies [125], larger phase II studies resulted in no detectable changes in CSF Aβ [126]. The recently disclosed phase III study results demonstrated no treatment effect for IVIG (Baxter press release 7 May 2103).

A more commonly used measure of target engagement in clinical studies employing immunotherapy, especially for those antibodies that recognize fibrillar Aβ, is amyloid PET imaging (for example, [102,103]). As with CSF, however, very few, if any, preclinical analyses describe a relationship between plaque reduction and functional efficacy. Any such analysis would then require overlay or inclusion of immunotherapy PK to be helpful in dose selection for clinical trials.

## Conclusion

The genetics and preclinical literature support the hypothesis that a 25% reduction in soluble Aβ is a scientifically based minimal criterion for any therapeutic directed toward lowering a soluble, pathologically relevant species of Aβ. Preclinical data demonstrate that soluble CSF Aβ can reflect soluble brain Aβ and PK/PD analyses of preclinical data reliably translate to the clinic for lowering of soluble CSF Aβ and, by inference, brain Aβ. While amyloid PET ligands can provide information on target engagement, especially for some antibodies, the relationship between plaque reduction, antibody exposure and efficacy has yet to be reported for any potential antibody therapeutic. The data from clinical trials disclosed to date suggest that no potential therapeutic has lowered soluble Aβ by 25%. Thus, while enormous progress has been made in understanding the basic mechanisms of AD and the identification of rational therapeutic mechanisms such as antibodies, GSIs, GSMs and BACEis, the amyloid hypothesis has yet to be adequately tested clinically by any of the current therapeutic moieties. Furthermore, the notion that the amyloid hypothesis is incorrect or has been disproven is premature. The potential of the current cohort of 'second generation' therapeutics, such as BACEis, which appear to provide potential for testing a broad range of Aβ lowering, and antibodies like crenezumab [127] and gantenerumab [128], is promising and may ultimately enable testing of the amyloid hypothesis.

## Abbreviations

Aβ: Amyloid-β; AD: Alzheimer’s disease; APP: Amyloid precursor protein; BACE: β-site APP-cleaving enzyme; BACEi: β-site APP cleaving enzyme inhibitor; cKO: Conditional knock out; CSF: Cerebrospinal fluid; FAD: Familial Alzheimer’s disease; GSI: γ-secretase inhibitor; GSM: γ-secretase modulator; KO: Knock out; MRI: Magnetic resonance imaging; NHV: Normal healthy volunteer; PD: Pharmacodynamic; PET: Positron emission tomography; PK: Pharmacokinetic; SILK: Stable isotope labeling kinetic; TgAPP: APP transgenic

## Competing interests

The authors are employees of Bristol-Myers Squibb.
